# Supplementary material for: One Size Fits All—Venomics of the Iberian Adder (Vipera seoanei, Lataste 1878) Reveals Low Levels of Venom Variation across Its Distributional Range
Source: Toxins (Basel). 2023 Jun 1;15(6):371. doi: 10.3390/toxins15060371 (PMC10301717; doi:10.3390/toxins15060371)

Figure S1. Whole venom profiles of the 49 *V. seoanei* specimens pooled to produce the reference proteome, under reducing conditions.

Horizontal dotted lines indicate the seeming molecular weight of polymorphic bands 1 to 10, as listed in Table S3. Localities of collection of the samples are reported. Venom sample 20VS016 was loaded twice because of the low quality of the profile obtained with the first electrophoretic run (code reported in grey).

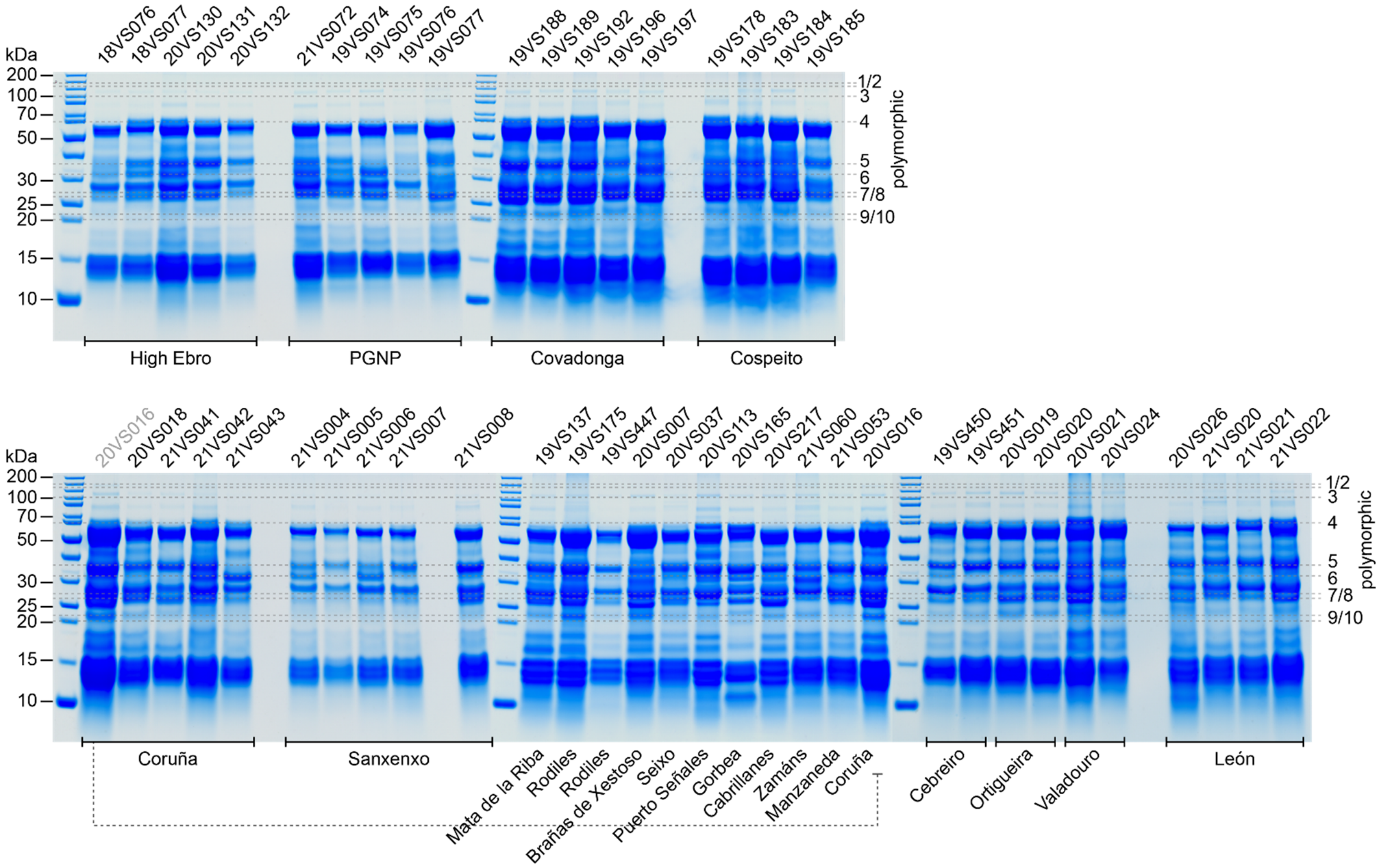

Supplement: Supplementary file 1 [file toxins-15-00371-s001.zip › Figure S1.pdf]
