# Supplementary material for: One Size Fits All—Venomics of the Iberian Adder (Vipera seoanei, Lataste 1878) Reveals Low Levels of Venom Variation across Its Distributional Range
Source: Toxins (Basel). 2023 Jun 1;15(6):371. doi: 10.3390/toxins15060371 (PMC10301717; doi:10.3390/toxins15060371)

Figure S2. Model predictions of occurrence of bands 1, 2, 4, 8, 9 and 10 in individual SDS-PAGE venom profiles in relation to the continuous predictors tested in single predictor GLMs.

The panels display the predicted probability of occurrence of: band 1 in relation to SVL (A); band 2 in relation to FOREST (B); band 4 in relation to AGRIC (C); band 8 in relation to SVL (D), BIO5 (E), and BIO12 (F); band 9 in relation to GEN2 (G); band 10 in relation to BIO5 (H) and FOREST (I).

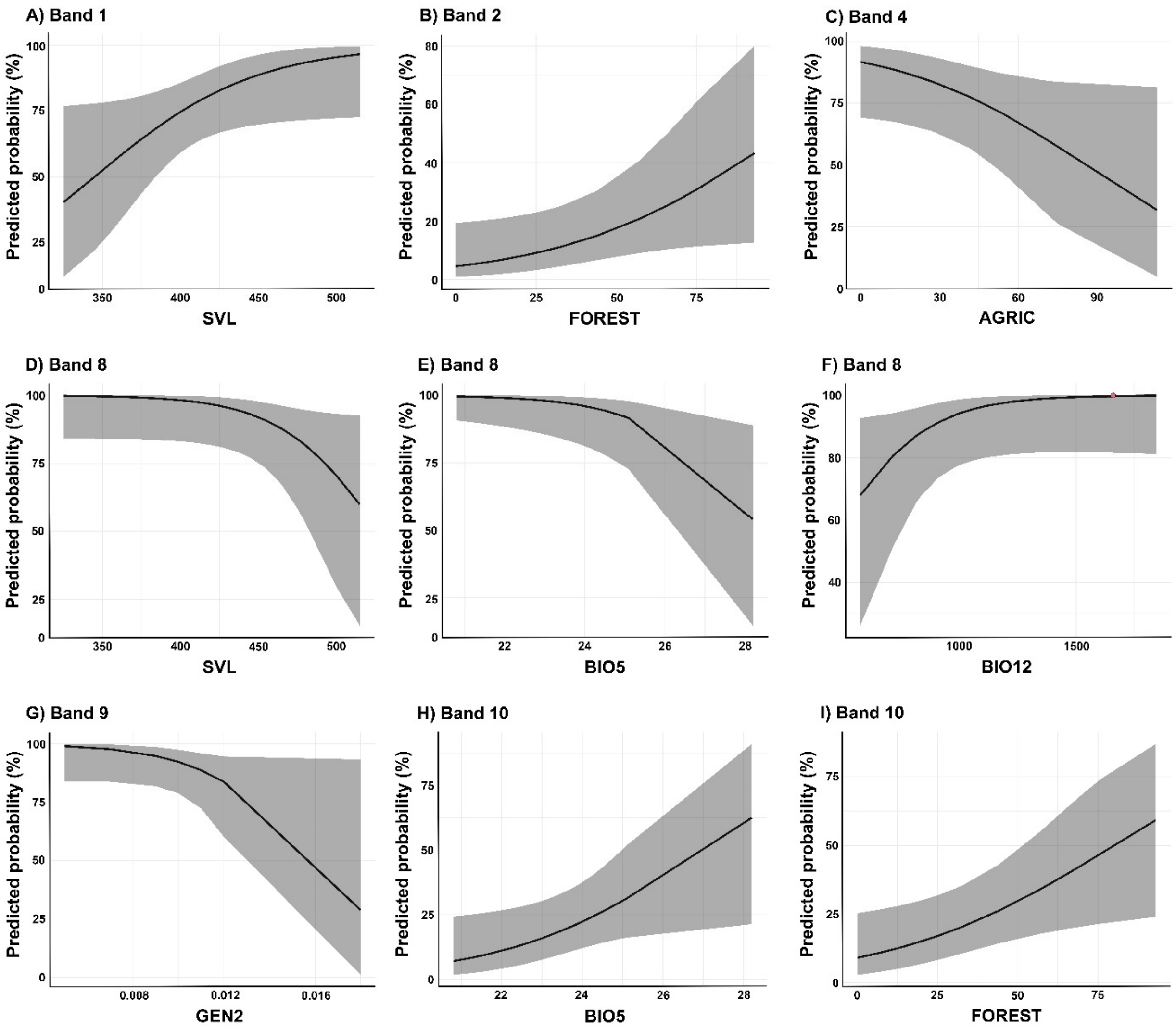

Supplement: Supplementary file 1 [file toxins-15-00371-s001.zip › Figure S2.pdf]
