# Supplementary material for: One Size Fits All—Venomics of the Iberian Adder (Vipera seoanei, Lataste 1878) Reveals Low Levels of Venom Variation across Its Distributional Range
Source: Toxins (Basel). 2023 Jun 1;15(6):371. doi: 10.3390/toxins15060371 (PMC10301717; doi:10.3390/toxins15060371)

Figure S3. The five geographically structured colour phenotypes currently recognised within *V. seoanei*.

Viper pictures modified from [48].

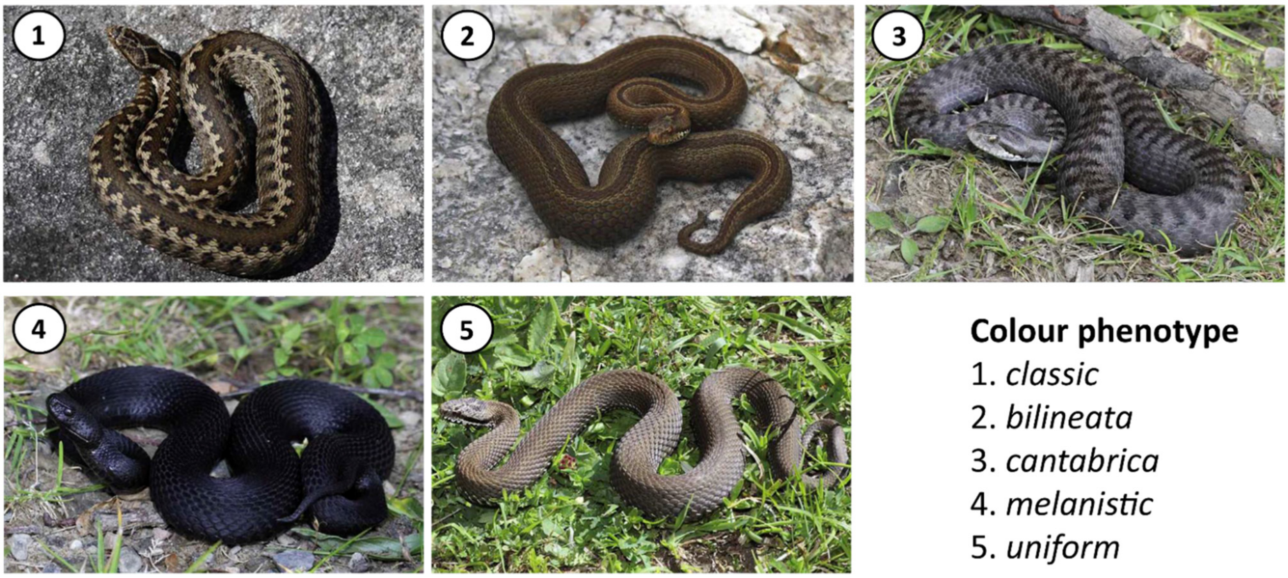

Supplement: Supplementary file 1 [file toxins-15-00371-s001.zip › Figure S3.pdf]
