# Supplementary material for: One Size Fits All—Venomics of the Iberian Adder (Vipera seoanei, Lataste 1878) Reveals Low Levels of Venom Variation across Its Distributional Range
Source: Toxins (Basel). 2023 Jun 1;15(6):371. doi: 10.3390/toxins15060371 (PMC10301717; doi:10.3390/toxins15060371)

Figure S4. Geographic genetic variation in *V. seoanei*.

First (a) and second (b) components of the Spatial Principal Component Analysis performed on the calculated genetic distances. Sampling localities are reported.

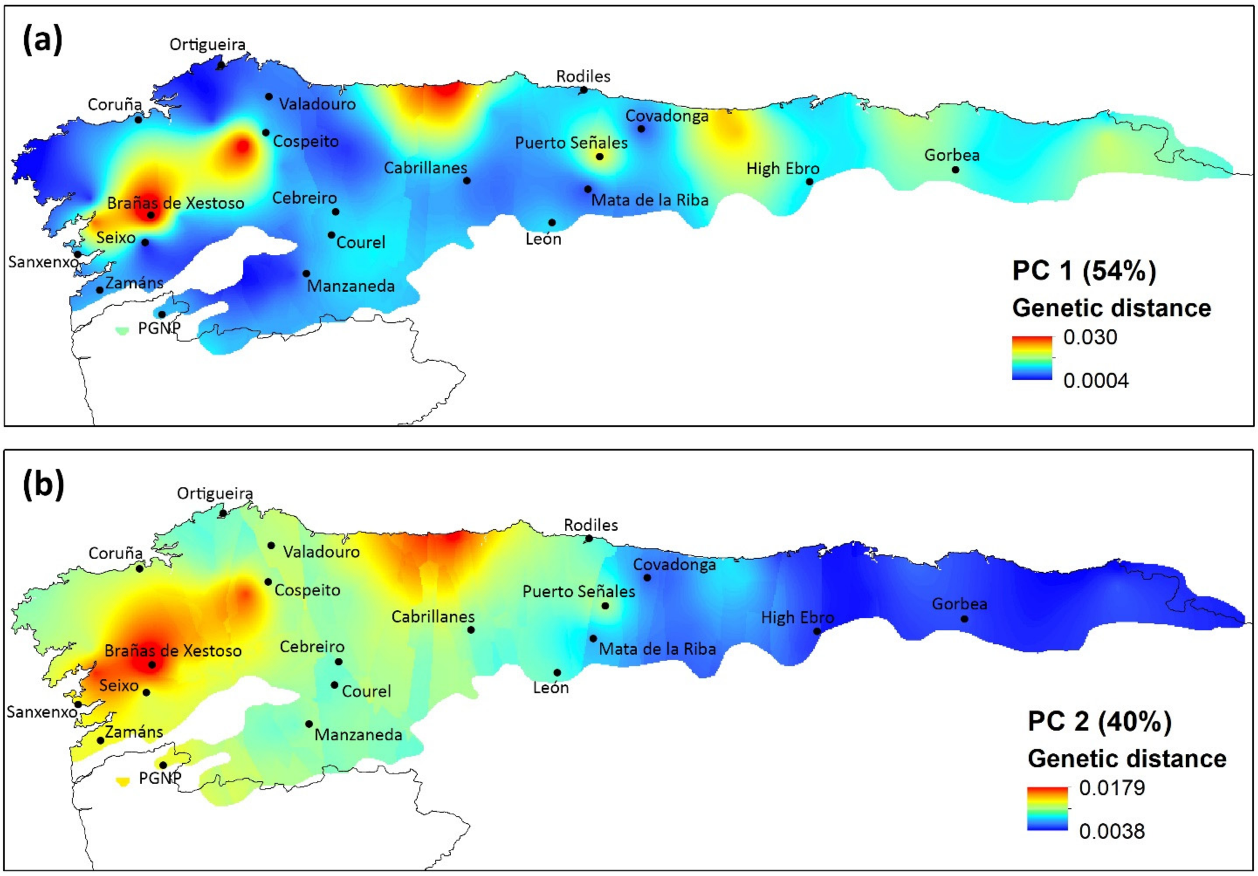

Supplement: Supplementary file 1 [file toxins-15-00371-s001.zip › Figure S4.pdf]
